# Highlights from the 2023 HRS/EHRA/APHRS/LAHRS expert consensus statement on practical management of the remote device clinic

**DOI:** 10.1016/j.hroo.2023.08.001

**Published:** 2023-08-28

**Authors:** Niraj Varma

**Affiliations:** Heart and Vascular Institute, Cleveland Clinic, Cleveland, Ohio

Implantation of cardiovascular implantable electronic devices (CIEDs) initiates an indefinite responsibility for follow-up, but the increasing volume of CIED implants poses significant management challenges. Remote monitoring (RM) promised a solution by reducing the necessity for in-person evaluations.^1^ However, this metric does not reflect the behind-the-scenes work undertaken to provide a remote management service. This goes largely unrecognized and insufficiently supported. Thus RM adoption and adherence has been fragmented.^2^ The 2023 international consensus statement not only maintains the strong 2015 recommendation for RM as a standard of care for CIED follow-up, but also identifies challenges and proposes practical solutions for the effective operation of an RM clinic.^1^^,^^3^ Some are highlighted subsequently.

Remote management requires patient education, enrollment, and scheduling, troubleshooting connectivity, data triage and review, alert management, documentation, communication, billing, and interfacing with electronic medical records. The task falls largely on allied health professionals but is often unstructured and underresourced, risking staff dissatisfaction and turnover. The 2023 consensus statement emphasizes that all members of the RM team should receive training, continuing education, and certification specific to RM and become knowledgeable about unique characteristics of different manufacturers’ devices and their associated RM platforms. Duties should be defined for each team member (ie, task based) who should operate in an organizational model adapted to each individual device clinic’s workflow. The service should be independent of industry, which should not participate directly in clinical care. It is essential that adequate time be dedicated to perform all RM tasks. The statement advocates for clinics to have a minimum of 3.0 full-time equivalents per 1000 patients on RM, comprising both clinical and administrative staff. Importantly, the service needs to be supported by administration to maintain minimal staff-patient ratios.

With an organizational model in place the device clinic can address priorities. Connectivity is the foundation for RM. While obvious, this lapses significantly in real-world practice (50% of RM capable device are not activated).^2^ The consensus statement indicates that RM enrollment should occur prior to postimplantation discharge from the hospital or clinic and be re-established promptly following generator change continuity. Management of disconnected patients is often onerous and time-consuming. Reasons may be patient or technology dependent. It is recommended that clinics have an established process to facilitate reconnection. Manufacturers have a role to notify patients directly about a disconnection and inform the clinic, providing technical support if needed. Additionally, manufacturers must contact clinics with details of safety advisories and provide clinic staff with adequate training, education, and technical support for their management.

Alert management is emphasized. Currently, unscheduled transmissions generate a considerable workload and are frustrating because most are clinically nonactionable. Careful programming customized to individual clinical indications may redress this. For instance, some repeating alerts can be deactivated safely (eg, atrial fibrillation alerts in patients with chronic atrial fibrillation, high burden of ventricular pacing alerts in patients with known atrioventricular block). In contrast, others are high priority (eg, ventricular shock therapies for patients with implantable cardioverter-defibrillators or for concerns related to device or lead function, low battery, and impedance out of range). Programming such a core set of alerts resulted in a low alert frequency (1 per patient-year) but with increased actionability (ie, a reduction in unnecessary notifications while maintaining early detection of important events).^4^ What is a reasonable time frame for response to such events? The document recognizes that most RM sites are not able to react immediately, or 24/7, and indicates that reaction time to red alerts should be within 1 business day.

Patients need to be integrated into the remote management program, receiving education about its function and the necessity to maintain connectivity, and not to regard it as an emergency notification system. Their concerns and expectations need to be addressed (digital literacy is not universal, evident with smartphone-based RM). Patients need to be informed about their clinic’s workflows and responsibilities. Remotely acquired data can be shared with patients according to preference (some patients want to know, while other patients just want reassurance that everything is okay). For patients not enrolled in an RM system, or in cases in which they have access only to facilities with limited on-site device interrogation capabilities, site-based remote interrogation technology can be placed in busy clinical areas for unscheduled interrogations (eg, urgent care centers).

It is recognized that not all clinics may be able to adhere to these recommendations. In such cases, vendor-neutral CIED management software (third-party platforms) may provide a route for streamlined remote management. Such systems obviate the need to concurrently manage each CIED manufacturer’s system separately. Alerts may be harmonized across all systems and customized to the needs of individual device clinics. The staff time required per device evaluation is reduced. However, patients need to be informed/consented for their data to be routed to a cloud-based system outside the hospital with accompanying cybersecurity risks. This needs to be balanced with the promise of improved patient outcomes.

An exciting look to the future comes from the recommendation for alert-based monitoring.^5^ This is a major paradigm shift. Over the years, device follow-up has transitioned from scheduled in-person appointments, through scheduled remote follow-ups, to remote monitoring combined with fewer in-person evaluations, which is the status today. Recent trials suggest that we can transition safely to continuous remote monitoring only (ie, conduct patient evaluations following alert notifications only when necessary). The requirements are reliable automatic continuous connectivity (only one manufacturer’s platform has been tested and proven in randomized trials) and restructuring device clinics to manage asynchronous (ie, not appointment-based) care. Reimbursement schemes will need to adapt. This approach promises a steep reduction in nonactionable patient evaluations (ie reducing unnecessary work) and redirecting valuable clinic resources to patients who develop problems (ie, seeing patients who need to be seen when they need to be seen [thus, exception-based care]). This model may be facilitated by processing by artificial intelligence of incoming data to stream actionable information regarding both CIED and disease status.

An underlying impediment to worldwide adoption of RM is the irregular availability of reimbursement. This international consensus document delivers a strong message to health care payors to provide adequate reimbursement, according to the country or regional health system, for costs of the RM system itself (hardware, software, and industry service); physician interpretation and effort of allied health professionals; and administrative and nonclinical personnel.

In summary, the 2023 consensus document is remarkable for the granularity of its guidance for implementing an effective remote device clinic and also for the number of new recommendations with a high proportion designated as “1-C EO,” reflecting the importance assigned by the well-seasoned authors on the basis of a rich collective experience, rather than on the basis of formal studies. We look forward to an era of clinics enabled to provide effective remote patient management realizing its potential for improved patient outcomes.
